# 

**Published:** 1893-11-25

**Authors:** 


PRICE TWOPENCE.

No. 374, Vol. XV.—Nov. 25th, 1893.

Registered at the General Post Office as a Newspaper for transmission in the United Kingdom and Abroad.]

NOVEMBER 25TH,
WITH EXTRA NURSING SUPPLEMENT.
Per Annum, 8s. 6d., post free.
Monthly Parts, 6d.; post free, 8d.
Ditto per Annum, 8s., post free.

### THE HOSPITAL

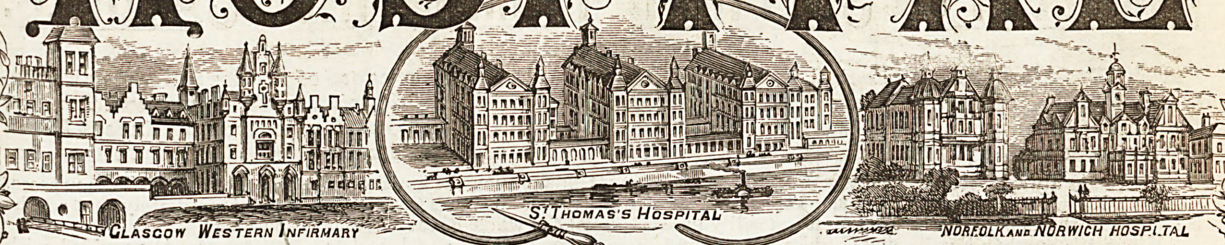

WITH EXTRA NURSING SUPPLEMENT.

No. 374. Vol. XV. SATURDAY, NOVEMBER 25TH, 1893. [TWOPENCE.

